# The Powers and Duties of the Board of Health

**Published:** 1912-01

**Authors:** Stephen Smith


### The Powers and Duties of the Board of Health.

BY STEPHEN SMITH, M. D.*

The Department of Health of the City of New York is an anomaly in municipal government. It has the power to make laws, to execute those laws and to sit in judgment on its own acts. Its acts within the sphere of its jurisdiction can not be interrupted or even reviewed by courts. The Board of Health is an "Imperium in imperio" so far as it stands related to the general city administration, and the civil courts.

The law creating the Health Department had its origin in the Citizens' Association in 1864-5. It was the joint product of its two committees—on public health and on law. As chairman of the committee on public health I made the first draft of the bill incorporating the sanitary provisions, and Hon. Dorman B. Eaton, as chairman of the committee on law, completed it with the legal provisions. The joint committees contained such men as Dr. Willard Parker, Dr. James R. Wood, William M. Evarts and Charles O'Connor. The question of investing the Board of Health with such arbitrary and autocratic powers was thoroughly discussed, both as to its necessity and as to its constitutionality.

There was a consensus of opinion that to be thoroughly equipped for all possible emergencies during the prevalence of epidemic pestilences a health authority should not be liable to be delayed in its action by the injunctions of courts, nor obstructed by the interference of any officials. The constitutionality of these provisions of the bill was the subject of a very critical discussion by the legal members of the committee, but the views of Mr. Eaton in favor of giving these extreme powers to the governing board of the department prevailed. He had recently made a critical examination of the English sanitary laws while in England and was impressed with the importance of clothing health authorities with extreme powers to enable them to meet promptly any emergency which might arise in their efforts to prevent or control epidemic diseases.

Such arbitrary powers to be exercised by a board of health was a new feature in legislation in this country and it was feared our Legislature would not pass the proposed bill. As a matter of fact in all the discussions of both houses on the merits of the bill this clause conferring great and unprecedented powers escaped criticism. This was due in whole or in part, probably, to the peculiarity of the phraseology of the section conveying these powers.

*The oldest member of the New York medical profession. From "Social Diseases," N. Y., Vol. No. 1, Report of Progress of the Movement for Their Prevention.

When, however, the measure as a law took effect and these powers began to be exercised, a large number of suits were begun in the courts, all based on the alleged unconstitutionality of the law. Great apprehensions were felt by the promoters of the law when on appeal the question of its constitutionality came in due course before the Court of Appeals. Mr. Eaton, as counsel of the board, conducted the case and the result was a decision in favor of the constitutionality of the law by a majority of one. The effect of this decision was immediate as regards the Metropolitan Board and far-reaching in its influence upon the future of sanitary administration in this country. The local inferior courts of the city, where the suits of the Board of Health are instituted, in the first instance, adopted rulings in accord with the decision of the Court of Appeals, and thus gave the Board of Health a most salutary prestige whenever the question of power or jurisdiction was the issue. A large number of suits of the Board against individuals maintaining nuisances which were being contested were discontinued owing to the immediate compliance of the defendants with the orders of abatement. From that time to the present the orders of the Board are regarded with profound respect by all classes of the community. Whenever the Board of Health declares officially any matter or thing, "A Nuisance Dangerous to Life and Detrimental to Health," and orders it abated, no citizen doubts its power to enforce its order in its own time and in its own way.

During my connection with the Board, 1868-1875, the great importance of such arbitrary powers, conservatively exercised, in the effective administration of health ordinances and orders, was repeatedly demonstrated. The Board was confronted with conditions affecting the public health so numerous as to be distracting, so hoary with age as to make it sacrilegious to condemn them, so protected by custom and privileges as to cause intense resentment at a suggestion of their abatement. Upwards of 200 slaughter houses lined the central streets and avenues, with inadequate drainage; droves of cattle, sheep and swine constantly traversed the best residential streets; 20,000 people lived in cellars, the ceilings being on a level with or below the street grade; the streets were lined with decomposing house refuse; sixty fat and bone boiling factories along the shores filled the air with sickening odors; the scavenger nightly gathered the contents of the open privies and carried his disgusting load for miles along the streets. Smallpox was epidemic and Asiatic cholera was approaching.

The Metropolitan Board proved equal to the enormous task suddenly thrust upon it. All its forces were quickly organized and set in motion. Every case of smallpox was removed to the hospital or quarantined at home; cleansing, disinfection, and vaccination followed, and this epidemic was soon under control. The first cases of cholera received equally prompt treatment and the pestilence never obtained a foothold.

The campaign against nuisances "dangerous to life and detrimental to health," many of which were as old as the city, was vigorously and relentlessly prosecuted until they were abated. The extreme measures resorted to where resistance to the orders of the Board was obstinate was the forcible removal of the cellar population, or "Troglodytes," as they were called, from their underground homes, to the sidewalks, and the tearing down and removal of the booths which surrounded Washington market.

The policy of the Board in the treatment of contagious and infectious diseases was based on the nature of the contagion and its known method of communication from one person to another. The life history of the contagious germ gave the clue to the methods of protection of the public health, from any individual disease. At that earlier period, quarantine of the infected person at home or in hospital, destruction or disinfection of infected materials and immunization of persons liable to be exposed to infection, were the practical applications of the policy of the Board. To this end the Board created hospitals for the care of the different forms of contagious diseases, provided apparatus for disinfection, and ambulances and vessels for the transportation of the infected. Though the efforts of the Board to enforce this policy have met with an opposition amounting to violence by ignorant and reckless people, yet the vast benefits which it confers both upon the public and the individual sick person are so apparent that not only is there little or no objection to compliance with the rules of the department, but even members of wealthy families suffering from communicable diseases seek the care and protection of these hospitals.

The duties of the Board of Health are strictly limited to the sphere of protecting and promoting the public health. Whatever matter, or thing, or condition, or circumstance, is "dangerous to life or detrimental to health," comes under its official cognizance and the law demands that it shall immediately proceed to apply the proper remedial measures. The Legislature not only placed no restrictions upon the Board's methods of procedure, but actually prevented any one from interfering with its operations. It virtually said to the Board, "Your duty is to protect the people of New York from every form of disease and promote the public health by any and every means which will surely accomplish that end." In such a mandate there is no sentimentalism, no question as to the kind of disease to be attacked, no query as to the sex of the person spreading infection, not even a caution as to what is the state of public opinion on this disease. It demands but one thing—results. By that test alone has the Board secured the confidence of the public, and by that test alone can it maintain that confidence. It never delays its operations until public opinion sustains it, but by its vigilance and success in discharging its duties, it creates a public opinion favorable to its methods and acts.

If in the light of the preceding discussion I were asked through what agency the public health is to be protected from the ravages of syphilitic and venereal diseases, I should not hesitate to reply emphatically, the Board of Health, and the Board of Health alone. For this duty it was created and for the prompt and effective accomplishment of that duty it was endowed with its arbitrary and autocratic powers. It has no discretion as to the nature of the disease from which the public is to be protected, nor its mode of communication, nor the sex, age or condition of the person or persons who are propagating it. The mandate of the Legislature is that the public health must be protected by the Board of Health.

How the Board is to solve the problem before it, must be determined in its own counsels. It is manifestly beset with many difficulties, if we consider the various methods adopted in foreign countries and at home. It is a question whether or not we approach the task in the right spirit. In the first place are we not too much dominated by foreign ideas and methods which practically result, whatever may be the underlying purpose, in protecting dissolute men by subjecting women to gross official treatment? The result is seen in the provisions of the Page law requiring the arrest of women on the public streets, their public trial, and commitment as vagrants or criminals—the whole scheme being most demoralizing to all parties witnessing it. Again, do we not allow our sentiments concerning the immoralities and criminalities of the sexes afflicted with these diseases to influence our judgment? We see this fact illustrated in our penal code which, from a medical view, contains many absurd and impractical provisions. Do not even the words prostitute, syphilis, venereal, prejudice our minds against persons and things in a way to affect our judgment?

My conclusion is that this matter in all of its details should be relegated to the Board of Health in order that it may be treated as a purely sanitary question.

Necessarily, the first inquiry would be to determine the extent of the diseases, that is, the morbidity, as appears not only in the acute stages, but in the chronic and obscure forms. This inquiry would disclose many important facts as to the classes most affected, their social relations, their habits of life. This inquiry would suggest many methods of procedure in reaching the active agents in propagating the diseases and bringing them under control. Especially serviceable would be inquiries in the vast number of public charities, hospitals and dispensaries. Here the work of actual control might begin by compelling inmates suffering from any form of these diseases to be cured before they are discharged. As an example of what a field for sanitary work is thus open for operations I may state that during my service in Bellevue Hospital many years ago a census was taken of the cases of syphilis and gonorrhea, and it was found that more than one-third of the patients were affected and a majority of them were males. These patients could have been detained until they were cured. There is no reason to believe that a census of the seventy-five existing hospitals would give results very widely different. The same is true of the fifty or more dispensaries. If this enormous population under control in public institutions could be systematically cured before they were discharged, nine-tenths of the battle would be won. In Massachusetts the law requires such cure to be made by the attending boards.

This is only a suggestion of the entrance of a sanitary authority into the field of protection of the public health against the social evil. Many clues to avenues of attack would develop as the work proceeded until these diseases would be a negligible quantity.

Doubtless the extermination of these diseases from the human family is impossible, as the method of propagation is beyond control, but that their prevalence may be immensely diminished by the combined action of all the health authorities of the entire country, municipal, State and national, there can be no doubt.

